# The codon-optimized Δ^6^-desaturase gene of *Pythium* sp. as an empowering tool for engineering *n*3/*n*6 polyunsaturated fatty acid biosynthesis

**DOI:** 10.1186/s12896-015-0200-6

**Published:** 2015-09-15

**Authors:** Sukanya Jeennor, Pattsarun Cheawchanlertfa, Sarinya Suttiwattanakul, Sarocha Panchanawaporn, Chanikul Chutrakul, Kobkul Laoteng

**Affiliations:** Bioprocess Technology Laboratory, Bioresources Technology Unit, National Center for Genetic Engineering and Biotechnology, National Science and Technology Development Agency, 113 Thailand Science Park, Khlong Nueng, Khlong Luang, Pathum Thani 12120 Thailand; Bioassay Laboratory, Bioresources Technology Unit, National Center for Genetic Engineering and Biotechnology, National Science and Technology Development Agency, 113 Thailand Science Park, Khlong Nueng, Khlong Luang, Pathum Thani, 12120 Thailand

## Abstract

**Background:**

The ∆^6^-desaturase gene, encoding a key enzyme in the biosynthesis of polyunsaturated fatty acids, has potential in pharmaceutical and nutraceutical applications.

**Results:**

The ∆^6^-desaturase gene has been isolated from a selected strain of Oomycetes, *Pythium* sp. BCC53698. The cloned gene (*PyDes6*) contained an open reading frame (ORF) of 1401 bp encoding 466 amino acid residues. The deduced amino acid sequence shared a high similarity to those of other ∆^6^-desaturases that contained the signature features of a membrane-bound ∆^6^-desaturase, including a cytochrome *b*_5_ and three histidine-rich motifs and membrane-spanning regions. Heterologous expression in *Saccharomyces cerevisiae* showed that monoene, diene and triene fatty acids having ∆^9^-double bond were substrates for PyDes6. No distinct preference between the *n-*3 and *n*-6 polyunsaturated fatty acyl substrates was found. The ∆^6^-desaturated products were markedly increased by codon optimization of *PyDes6*.

**Conclusion:**

The codon-optimized ∆^6^-desaturase gene generated in this study is a promising tool for further reconstitution of the fatty acid profile, in a host system of choice, for the production of economically important fatty acids, particularly the *n-*3 and *n*-6 polyunsaturated fatty acids.

**Electronic supplementary material:**

The online version of this article (doi:10.1186/s12896-015-0200-6) contains supplementary material, which is available to authorized users.

## Background

Polyunsaturated fatty acids (PUFAs) are important metabolites, which have benefits on human and animal health. Besides being a metabolic fuel, they also play crucial roles in membrane biology and signaling processes in living cells [1–3]. As a consequence, the demand for PUFAs has continually increased in recent years. Although PUFAs are widely distributed in natural resources, such as plant seed, fungi and marine organisms [4], the search for economical and renewable resources of PUFAs has been extensively persued due to a concern for either an insecurity of supply or healthier performance of PUFA products. Metabolic engineering of the PUFA biosynthetic pathway has been of considerable interest as an alternative approach to produce biomass rich in specific PUFAs [5, 6]. However, this modern technology requires potent genes involved in relevant metabolic pathways.

PUFA biosynthesis is generally associated with a set of membrane-bound enzymes, namely fatty acid desaturase and elongase, which catalyze the introduction of a double bond and the 2-carbon chain extension of fatty acids, respectively. These enzymes have different specificities on fatty acid substrates, relating to acyl chain length and double bond position [7]. ∆^6^-Desaturase is a key enzyme involved in the biosynthesis of *n*-3 and *n*-6 PUFAs, which is responsible for the conversion of essential fatty acids, linoleic acid (LA, C18:2∆^9,12^) and α-linolenic acid (ALA, C18:3 ∆^9,12,15^) to γ-linolenic acid (GLA, C18:3∆^6,9,12^) and stearidonic acid (STA, C18:4∆^6,9,12,15^), respectively. Subsequently, the desaturated C18 PUFAs can be further metabolized to longer-chain PUFAs, such as arachidonic acid (ARA, C20:4∆^5,8,11,14^) and eicosapentaenoic acid (EPA, C20:5∆^5,8,11,14,17^), through alternating series of desaturation and elongation. The highly unsaturated fatty acid, EPA, is one of the nutritionally important *n*-3 PUFAs, which can be synthesized via either *n*-3 or *n*-6 pathways. To manipulate the oil composition of organisms of choice, by a metabolic engineering approach, a high expression level of the ∆^6^-desaturase enzyme in a heterologous host is required. As well as optimized culture conditions, an efficient promoter and substrate availability, codon optimization is one of the most common approaches for improving heterologous gene expression in some host organisms [8–13]. Although genes encoding for ∆^6^-desaturase have been cloned and characterized from various organisms [14–20], research on codon optimization has been limited [13].

Very recently we employed *Pythium* sp. BCC53698 as a genetic resource for the isolation of the ∆^6^-elongase gene, based on its fatty acid profile [21]. This Oomycete fungus synthesizes ARA and EPA as the end products of *n*-6 and *n*-3 PUFAs, respectively, indicating that it would be a potential source for the ∆^6^-desaturase gene. In this work, we have identified and functionally characterized the ∆^6^-desaturase gene of *Pythium* sp. BCC53698. Substrate specificity and preference were investigated by heterologous expression in *S. cerevisiae*. Codon optimization of the ∆^6^-desaturase gene was also performed to enhance the product yield.

## Results

### Identification and characterization of the *Pythium* ∆^6^-desaturase gene

The gene coding for ∆^6^-desaturase was cloned from *Pythium* sp. using PCR technology. A 700-bp fragment was obtained, and its deduced amino acid sequence showed a high sequence similarity to ∆^6^-desaturases of other organisms. Inverse PCR and RACE techniques were then performed to obtain the full-length cDNA. Under the optimized PCR conditions (annealing temperature of 55 °C) the product targets approximately 550 and 400 bp in length, were derived from inverse PCR and 5′-RACE, respectively. Taken together, the full-length *PyDes6* gene contained an ORF of 1401 bp encoding 466 amino acid residues with a calculated molecular mass of 52.8 kDa. The deduced amino acid sequence of *PyDes6* shared the highest homology with Oomycete ∆^6^-desaturases, which had 68 % identity with the functionally characterized ∆^6^-desaturase from *Phytophthora infestans* [22] and *Pythium splendens* [23].

**Fig. 1:**
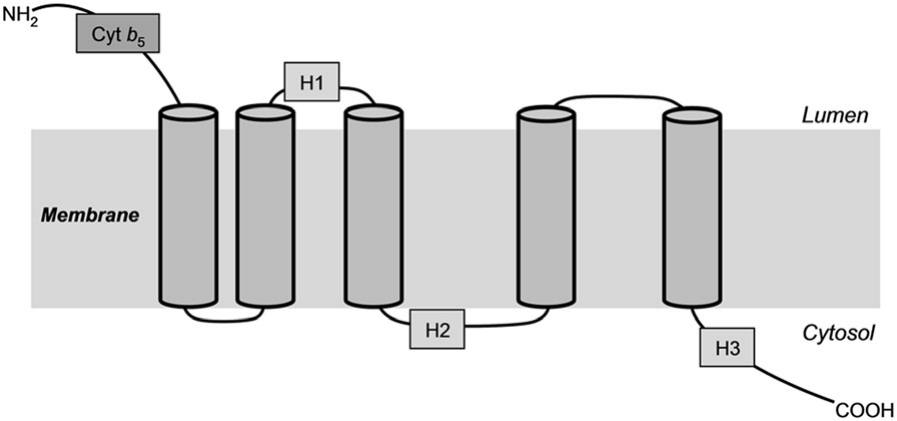
Topology model of PyDES6. Enzyme topology was predicted based on TMHMM and Phobius programs. The cylinders represent transmembrane regions of the enzyme, Cyt*b*_5_ indicates the N-terminal cytochrom *b*_5_ domain, and three histidine-rich motifs are represented by H1, H2 and H3

The *PyDes6* sequence contained a conserved characteristic of membrane-bound desaturases, which included three histidine-rich motifs (HXXXH, HXXHH and QXXHH). In addition, the cytochrome *b*_5_-like motif (HPGG) was found at its N-terminus as shown in Additional file 1: Figure S1. Hydropathy analysis of the *Pythium* desaturase revealed five hydrophobic regions (Fig. 1). All histidine-rich motifs were present in the hydrophilic portion that might be a location at the cytoplasmic surface of the membrane. These features coincide with a model of the topology of membrane-bound desaturases [24]. The phylogenetic tree revealed that PyDES6 belongs to the ∆^6^-desaturase of Oomycetes (Additional file 1: Figure S2) close to the subgroup of marine algae. This result is in agreement with the fatty acid profile, which is used as a chemotaxonomic marker, showing that *Pythium* sp. accumulates a *n*-3 long-chain PUFA (EPA) similar to some marine algae, such as *Nannochloropsis oculata* [25] and *Phaeodactylum tricornutum* [26]. These results suggest that this gene encodes a putative ∆^6^-desaturase, which might be responsible for the introduction of ∆^6^-double bond into acyl chains.

### Functional analysis of the *Pythium* ∆^6^-desaturase

**Fig. 2:**
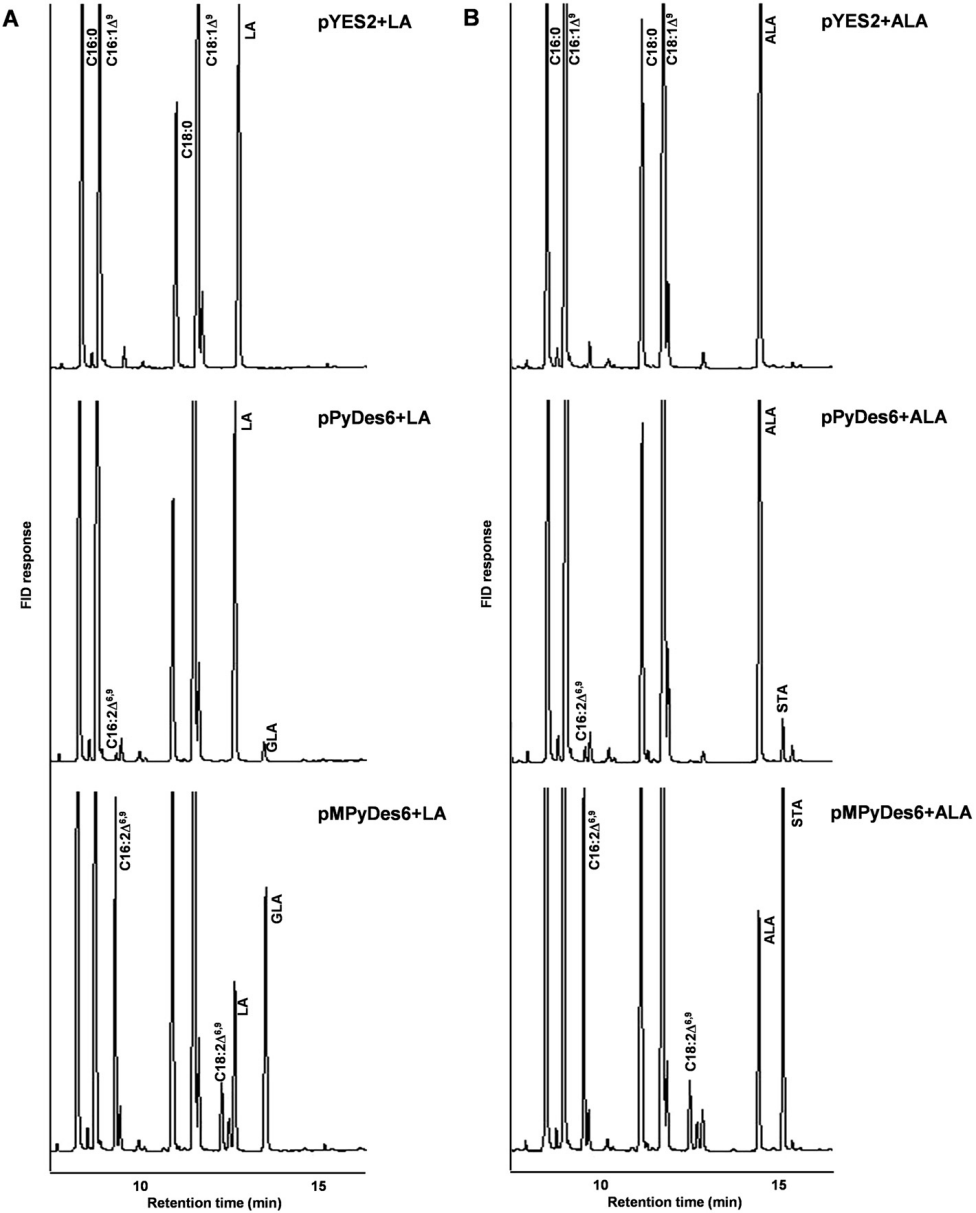
Chromatographic profiles of the yeast transformants carrying pYES2 (upper), pPyDes6 (middle) and pMPyDes6 (lower) plasmids. The transgenic yeasts were cultured in the presence of exogenous fatty acid substrates, LA (**a**) and ALA (**b**), and the gene expression was induced by addition of 2 % (w/v) galactose

To verify the function of the cloned *Pythium* desaturase, heterologous expression in *S. cerevisiae,* under the control of *GAL1* promoter, was performed. The transformed yeast cells were supplemented with linoleic acid (C18:2∆^9,12^, LA) as a fatty acid substrate. After the induction of gene expression fatty acid analysis revealed an extra peak, with the retention time corresponding to γ-linolenic acid (C18:3 ∆^6,9,12^, GLA), was detected in the yeast transformants carrying the *PyDes6*, which was absent in the yeast containing empty vector pYES2 (Fig. 2 and Table 1). This result confirmed that *PyDes6* encodes for ∆^6^-desaturase that catalyzes the insertion of double bond into LA yielding GLA.

**Table 1:** Fatty acid composition of yeast transformants containing the empty plasmid (pYES2) and recombinant plasmids (pPyDes6 and pMPyDes6) grown in SD medium supplemented with 50 μM fatty acid substrates

Yeast transformant	Fatty acid composition in total fatty acids (% w/w)									
	C16:0	C16:1∆^9^	C16:2∆^6,9^	C18:0	C18:1∆^9^	C18:2∆^6,9^	C18:2∆^9,12^ (LA)	C18:3∆^6,9,12^ (GLA)	C18:3∆^9,12,15^ (ALA)	C18:4∆^6,9,12,15^ (STA)
pYES2 (control)										
No fatty acid addition	22.1 ± 0.4	43.7 ± 0.5	-	7.5 ± 0.1	26.7 ± 0.2	-	-	-	-	-
+LA	23.5 ± 0.3	34.3 ± 0.9	-	8.5 ± 0.1	20.2 ± 0.7	-	13.6 ± 1.2	-	-	-
+ALA	22.7 ± 0.3	32.8 ± 0.3	-	8.7 ± 0.1	20.9 ± 0.6	-	-	-	14.9 ± 0.7	-
pPyDes6										
No fatty acid addition	19.1 ± 1.3	43.7 ± 3.6	1.4 ± 0.1	7.9 ± 0.5	27.8 ± 1.9	-	-	-	-	-
+LA	22.4 ± 0.5	37.6 ± 0.4	0.2 ± 0.0	7.1 ± 0.2	21.1 ± 0.3	-	11.2 ± 0.0	0.6 ± 0.0	-	-
+ALA	21.0 ± 2.1	29.9 ± 8.1	0.2 ± 0.1	5.8 ± 2.0	28.9 ± 11.4	-	-	-	13.8 ± 1.0	0.6 ± 0.2
pMPyDes6										
No fatty acid addition	22.6 ± 0.2	31.9 ± 0.1	11.4 ± 0.2	7.7 ± 0.0	23.3 ± 0.1	3.1 ± 0.0	-	-	-	-
+LA	24.8 ± 0.2	26.6 ± 0.0	5.7 ± 0.3	9.6 ± 0.1	20.8 ± 0.0	1.6 ± 0.0	4.1 ± 0.1	6.9 ± 0.1	-	-
+ALA	24.6 ± 0.3	25.2 ± 0.2	5.9 ± 0.0	10.3 ± 0.1	20.3 ± 0.4	1.6 ± 0.1	-	-	4.7 ± 0.3	7.3 ± 0.1

Substrate utilization of PyDes6 was investigated by feeding the yeast transformants with fatty acids with different chain lengths and double bond positions, including saturated and monounsaturated fatty acids and PUFAs. In addition to LA substrate, PyDes6 could catalyze the ∆^6^-desaturation of *n*-3 PUFA, α-linolenic acid (C18:3∆^9,12,15^, ALA), yielding stearidonic acid (C18:4∆^6,9,12,15^, STA) (Fig. 2 and Table 1). In addition, the ∆^6^-desaturated 16C fatty acid (C16:2∆^6,9^) was detectable indicating that the endogenous monoene fatty acid (C16:1∆^9,^) was a substrate for PyDes6. However, the endogenous and exogenous saturated fatty acids tested were not desaturated. Considering substrate preference, there was not much difference between the conversion rates of LA (5.4 %) and ALA (4.2 %) to the respective ∆^6^-desaturated products (Table 2).

**Table 2:** Conversion rate of fatty acyl substrates of yeast transformants containing the recombinant plasmids (pPyDes6 and pMPyDes6)

Substrate	Conversion rate of substrate to product (%)	
	pPyDes6	pMPyDes6
C16:1∆^9^	3.2 ± 0.0	26.3 ± 0.2
C18:1∆^9^	nd^a^	11.8 ± 0.1
LA	5.4 ± 0.0	62.7 ± 0.6
ALA	4.2 ± 1.8	60.9 ± 1.3

^a^ *nd* not detectable

### Influence of codon optimization on *Pythium* ∆^6^-desaturase activity

Codon usage of the *PyDes*6 for its expression in fungal system was optimized. Using the OptimumGene™ algorithm, 21.1 % of the 1401-bp coding region was changed, which led to 56.9 % codons being optimized. The GC content was reduced from 64.9 % to 51.9 %. Codon adaptation index (CAI) of the optimized desaturase gene (*MPyDes*6) was increased from 0.5 to 0.9, which is regarded as good in terms of high gene expression in the desired expression organism. The full-length cDNA of *MPyDes*6 was ligated to pYES2 vector under the control of *GAL1* promoter, generating the pMPyDes6 plasmid. Heterologous expression of the codon-optimized desaturase showed that the MPyDes6 retained the function of ∆^6^-desaturase, which could convert LA and ALA to GLA and STA, respectively (Fig. 2 and Table 1). It could also convert endogenous monoene fatty acids, C16:1∆^9^ and C18:1∆^9^ to hexadecadienoic acid (C16:2∆^6,9^) and octadecadienoic acid (C18:2∆^6,9^), respectively, whereas only C16:2∆^6,9^ was slightly accumulated in the pPyDes6 transformant. Compared with the native enzyme (PyDes6), the conversion of LA to GLA by the MPyDes6 transformant sharply increased from 5.4 to 62.7 %. Similarly, the higher conversion rate of ALA (60.9 %) was found in the transformant carrying the codon-optimized desaturase (Table 2). Thus, the increase of ∆^6^-desaturated products was a result of codon optimization showing the effective approach for enhancement of the PUFA production in fungal system. The yeast transformants with the empty vector (control), native (*PyDes6*) and codon-optimized (*MPyDes6*) genes did not show difference in the cell growth and biomass (Additional file 1: Figure S3).

## Discussion

The biosynthesis of long-chain PUFAs through a series of desaturation and elongation, the ∆^6^-desaturase has been documented to be a rate-limiting enzyme, and its expression is regulated by several factors [27]. Among ∆^6^-desaturases from diverse organisms, there is a differentiation in catalytic activity in terms of utilization of acyl substrates that might be a result of genetic variation. The *PyDes6* gene identified from this work showed conserved characteristics of membrane-bound desaturases, including a cytochrome *b*_5_ and three histidine-rich motifs, and transmembrane domains [28]. It has been reported that the cytochrome *b*_5_ motif contributes as an electron donor in the electron transport system of fatty acid desaturation by forming the core of a heme binding domain [29]. The histidine-rich motifs are known to be the catalytically essential residues [30].

From the study of substrate specificity, the *Pythium* enzyme was specific to 16C and 18C fatty acid substrates having ∆^9^-double bond. Thus, we classified the PyDes6 into the front-end desaturase family, which catalyzes the addition of a double bond between the pre-existing double bond and the carboxyl end of PUFAs [28]. Although the structural and functional characteristics of PyDes6 shared common features of ∆^6^-desaturases for catalyzing PUFA synthesis, the discrimination in substrate preference was found. Interestingly, both *n*-3 (ALA) and *n*-6 (LA) PUFAs having ∆^9^-double bond were substrates for PyDes6 enzyme at a similar level of substrate conversion rate in contrast to *Phytophthora* ∆^6^-desaturase, which prefers LA over ALA [22]. In the plant *Primula*, ALA was a preferred substrate for the ∆^6^-desaturase [31].

The synthesis of long-chain PUFAs is derived from either the *n*-3 or *n*-6 pathway. The capability of PyDES6 to utilize the *n*-6 fatty acid (LA) and *n*-3 fatty acid (ALA) at a similar levels, facilitates the application of this gene for engineering of PUFA pathway of choice in a broad range of organisms (plants and microorganisms). The outcome will depend on substrate availability or the predominant fatty acid accumulated in the host cells. The low proportion of ∆^6^-desaturated products (GLA and STA) found in the yeast transformant might be a result of a difference in codon usage between *Pythium* and *S. cerevisiae*. This could be explained by the evolutionary relationship of the ∆^6^-desaturase gene derived from *Pythium* which is closer to marine algae and diatoms than other fungi. This phenomenon is consistent with the taxonomic classification of fungal-like Oomycetes [32].

To enhance the production yields in engineered strain, the optimization of codon usage complemented to a host machinery of target was implemented in this study. The significant increase (*P* < 0.05) in the substrate conversion rate observed in the yeast harboring codon-optimized gene was presumably derived from an improved translation efficiency in the host system. The increased GLA content obtained by expressing the codon-optimized ∆^6^-desaturase of *Pythium* in *S. cerevisiae* was relatively higher than the yeast cultures carrying ∆^6^-desaturases of other organisms [31, 33, 34]. These results suggest that codon usage of the host organism had a profound effect on the expression of *Pythium* enzyme. Additionally, substrate availability is also an important criterion for increasing the production yield of PUFAs, which can be achieved either through genetic or physiological manipulation.

The low lipid content found in *S. cerevisiae* [35] is seen as a limitation to its use for high yield lipid production on a large scale. Consequently the development of other known oleaginous strains is key to our target in the exploitation of metabolic engineering to develop organisms which deliver high product yield.

## Conclusions

This study describes the cloning of a ∆^6^-desaturase gene from *Pythium* sp. The gene encoded an enzyme which catalyzed the ∆^6^-desaturation of the fatty acyl substrates having ∆^9^-double bond. The product yields were markedly enhanced by codon optimization of the *Pythium* gene. The redundancy in substrate utilization of the enzyme the codon-optimized gene could be exploited as potential genetic tool for production of nutritionally important PUFA(s) by reconstituting fatty acid profile in biological systems of commercial interest through *n*-3 or *n*-6 pathway.

## Methods

### Strains and growth conditions

*Pythium* sp. BCC53698, deposited in the BIOTEC culture collection (BCC), National Center for Genetic Engineering and Biotechnology, was used as the genetic resource for the isolation of a ∆^6^-desaturase gene. The fungus was grown in a semi-synthetic medium [36] at 30 °C to logarithmic phase. *S. cerevisiae* DBY746 (*MAT*α, *his*3-∆1, *leu*2-3, *leu*2-112, *ura*3-52, *trp*1-289) was employed as a host for functional analysis of the cloned gene. The yeast was generally grown in a complete medium, YPD (1 % bacto-yeast extract, 2 % bacto-peptone and 2 % glucose). For transformant selection, a minimal medium, SD (0.67 % bacto-yeast nitrogen base without amino acids and 2 % glucose) supplemented with 20 mg/l L-tryptophane, 20 mg/l L-histidine-HCl and 30 mg/l L-leucine was used. *Escherichia coli* DH5α was used for plasmid propagation.

### Nucleic acid manipulation

Genomic DNA from *Pythium* sp. was extracted from young mycelia (16-hr culture) using the protocol modified from Raeder and Broda [37]. Total RNA was extracted using TRI reagent (Molecular Research Center, Inc., Ohio) according to manufacturer’s instruction. First-strand cDNA was synthesized using P29-oligo-dT-AP primer (Table 1) and SuperScript II first-strand synthesis system (Invitrogen, CA). Then, approximately 50 ng of the first-strand cDNA were further used as templates for 5′-RACE.

### Cloning of full-length cDNA of ∆^6^-desaturase from *Pythium* sp.

To clone the gene coding for *Pythium* ∆^6^-desaturase (*PyDes*6), a portion of gene was amplified by PCR using a genomic DNA template and degenerate primers, P14-PyDes6-F and P15-PyDes6-R (Table 3), which were designed based on conserved amino acid sequences of ∆^6^-desaturases of several fungi, F(W/Y)QQSGWLAH and (Q/N)YQ(I/V)(E/D)HHLFP, respectively. The reaction was carried out as follows; an initial denaturation step at 94 °C for 3 mins; followed by 35 cycles at 94 °C for 35 s; primer-specific annealing temperature for 40 s and 72 °C for 1 min; and a final extension step of 72 °C for 5 mins. The expected PCR fragment (700 bp) was subcloned using TOPO TA cloning kit (Invitrogen, CA) following the manufacturer’s instructions. Plasmids were then extracted and purified using the QIAprep mini kit (Qiagen), and sequenced.

**Table 3:** Oligonucleotide primers used for amplifying ∆^6^-desaturase gene of *Pythium* sp.

Primer name	Oligonucleotide sequence (5′-3′)	Strand^a^
P29-oligo-dT-AP	GGCCACGCGTCGACTAGTACTTTTTTTTTTTTTTTTT	-
AAP	GGCCACGCGTCGACTAGTACGGGIIGGGIIGGGIIG	+
AUAP	GGCCACGCGICGACTAGTAC	+
P14-PyDes6-F	TTYTGRCARCARTCIGGITGRYTIGCICAYGA	+
P15-PyDes6-R	GGRAAIARRTGRTGYTCDATYTGRTARTT	-
P89-PyDes6-R	TTGACGTCGCCGACGAGG	-
P91-PyDes6-RN	TAGTAGTGGTCGTCCCAGAC	-
P96-PyDes6-RN	GAGGCCCTTGACCTTGACG	-
P92-PyDes6-F	AGCAGTTCGCCTTCGGTCG	+
P93-PyDes6-FN	TGTACGCCAACATGTCCCTG	+
P94-PyDes6-FN	GCAACATCACGCCGAGTCTC	+
P99-PyDes6-BamHI-F	CGCGGATCCATGGTGGACCCCAAGCCC	+
P100-PyDes6-EcoRI-R	CCGGAATTCCTACATGGCCGGGAACTCGG	-

^a^Sense and anti-sense strands are denoted by plus (+) and minus (-) symbols, respectively

The inverse PCR and RACE techniques were used for cloning of the full-length *PyDes6* gene. According to the derived DNA sequences of the PCR product, six gene-specific primers (Table 3) were designed for amplification of 3′- and 5′-cDNA ends. For inverse PCR, 150 ng of genomic DNA were digested with *Bam*HI (Thermo scientific) in a final reaction volume of 30 μl. The reaction was incubated for about 1–2 h at 37 °C and then inactivated by heating at 80 °C for 15 mins. Digested genomic DNA was purified using the GenepHlow Gel/PCR kit (Geneaid) and circularized by self-ligation with T4 ligase as recommended by the manufacturer (Promega). The reaction was incubated at 4 °C for 16 h [38]. Subsequently, inverse PCR reaction was set by using the self-ligated DNA as a template and the combination of appropriate primer pairs (Table 3) in a final volume of 25 μl. Thermal cycles were as follows; denaturation at 94 °C for 3 mins; followed by 35 cycles of 94 °C for 40 s; 55 °C for 45 s and 72 °C for 2 mins; and the final extension step of 72 °C for 7 mins. To obtain the 5′-end cDNA fragment, the 5′-RACE technique was carried out using 5′-RACE cDNA amplification kit (Invitrogen, CA). PCR was performed using the AAP primer (Table 3) and antisense primer (P89-PyDes6-R). Nested PCR was also conducted to derive a specific product using P91-PyDes6-RN and P96-PyDes6-RN primers. All PCR fragments were purified using the GenepHlow Gel/PCR kit (Geneaid) following the manufacturer’s protocol and were then subcloned into pGEM-T easy vector (Promega, USA) for further sequencing using a service of Macrogen (Korea). The sequences obtained were analyzed against known nucleotide or amino acid sequences available in NCBI GenBank database using the BLAST program (http://blast.ncbi.nlm.nih.gov/). The cDNA sequence of *PyDes6* has been deposited in GenBank and assigned the accession number of KM609327

### Structural characterization and phylogenetic analysis

Multiple amino acid alignments of *PyDes6* and the ∆^6^-desaturases of other organisms were performed by using the ClustalW [39] and GeneDoc programs [40]. Transmembrane regions were predicted by the TMHMM algorithm [41] and Phobius [42]. An unrooted phylogenetic tree was constructed based on alignment of amino acid sequences using the neighbour-joining method in MEGA5 software [43]. A total of 1000 bootstrap tests were sampled to determine the confidence in each node on the consensus tree.

### Codon optimization of the *Pythium* ∆^6^-desaturase

The coding region of the *PyDes6* was optimized based on the general rule of RNA stability and the codon usage of the host system. In this study, the OptimumGene TM algorithm was implemented for optimizing a variety of parameters, which are critical to the efficiency of *PyDes6* expression in fungal system. The DNA fragment coding for the optimized codon of *PyDes6* was synthesized by a service of Genscript (Piscataway, USA). The sequence of *MPyDes6* has been deposited in GenBank and assigned the accession number of KT438838. The *Bam*HI and *Eco*RI sites were incorporated at the start and stop codons, respectively, to further facilitate subcloning into the expression vector, pYES2. The fragment was located downstream of the *GAL1* promoter yielding pMPyDes6 plasmid.

### Heterologous expression of native and codon-optimized *PyDes6* cDNAs in *S. cerevisiae*

For functional analysis of the native and codon-optimized gene in the heterologous host, the native *PyDes6* cDNA fragment was amplified by RT-PCR using high fidelity *Taq* polymerase (Invitrogen, CA) with the specific primers, P99-PyDes6-*Bam*HI-F and P100-PyDes6-*Eco*RI-R (Table 3) that contained *Bam*HI or *Eco*RI sites, respectively, to facilitate subsequent cloning. The amplified product was subcloned into pYES2 expression vector (Invitrogen, CA) downstream of the *GAL1* promoter to generate pPyDes6 plasmid. The recombinant plasmids (pPyDes6 and pMPyDes6) and the empty plasmid (pYES2) were then individually transformed to *S. cerevisiae* using PEG/lithium acetate method following the manufacturer’s protocol (Invitrogen, CA). Transformed cells were selected by uracil prototrophy on SD medium lacking uracil. The yeast transformants were then grown in SD medium containing 2 % (w/v) raffinose and 50 μM of individual fatty acids (Sigma, St. Louis, MO) at 30 °C for 48 h. These fatty acids included saturated fatty acids (myristic acid; C14:0, pentadecanoic acid; 15:0, margaric acid; C17:0, nonadecanoic acid; C19:0, arachidic acid; C20:0 and behenic acid; C22:0), monounsaturated fatty acid (myristoleic acid; C14:1∆^9^, *cis*-vaccenic acid; C18:1∆^11^,*cis*-eicosenoic acid; C20:1∆^11^ and erucic acid; C22:1∆^13^) and PUFAs (LA and ALA). Subsequently, gene expression was induced by adding galactose to a final concentration of 2 % (w/v), and further cultivated for 48 h at 25 °C. Cell growth was determined by spectrophotometry with absorbance at 600 nm (OD_600_). Cells were harvested by centrifugation, washed twice with 0.1 % tritonX 100, dried to derive a constant weight. Three independent experiments were carried out for each culture. The rate of substrate conversion was calculated as follows; percentage of conversion rate = [product formed/(substrate + product formed)] × 100. Comparison of the substrate conversion rates between the yeast transformants carrying the native and codon-optimized gene was performed to determine the efficiency and substrate preference. The statistical analysis of substrate conversion rates was done using the program SPSS 11.5.

### Fatty acid analysis

To determine the fatty acid composition of the yeast transformants, fatty acid methyl esters (FAMEs) were prepared using the method modified from Lepage and Roy [44]. The dried yeast cells were directly transmethylated with 2 ml of 5 % HCl in methanol at 80 °C for 90 mins. After the samples were cooled to room temperature, 1 ml of distilled water and 1 ml of 0.01 % (v/v) butylated hydroxytoluene (BHT) in *n*-hexane were added and the suspensions were shaken vigorously. The *n*-hexane phase containing FAMEs was collected and dried under N_2_ stream. Then, the samples were resuspended in 100 μl hexane and analyzed by gas chromatography using a GC-17A gas chromatograph (Shimadzu, Tokyo) equipped with a capillary column Type OMEGAWAX™250 (Supelco, USA) (30 m × 0.25 μm) and a flame ionization detection. Helium was used as a carrier gas at a constant flow rate of 1.0 ml⋅min^-1^. The column and detector temperatures were set at 150–230 °C and 260 °C, respectively. Area measurements of the chromatographic peaks were used to calculate the relative amount of the individual fatty acids. FAMEs were identified by reference to the retention time of FAME standards (Sigma, St. Louis, MO).

## Additional file

Additional file 1: Figure S1. Comparison of deduced amino acid sequences between PyDes6 of *Pythium* sp. BCC53698 and other ∆^6^-desaturases, including *P. infestans* (PinDES6), *P. splendens* (PspDES6), *N. oculata* (NoDES6), *M. alpina* (MaDES6)*, M. rouxii* (MrDES6) and *Borage officinalis* (BoDES6). The conserved histidine boxes and cytochrome *b*_5_ heme-binding motif are underlined and boxed, respectively. **Figure S2.** Phylogenetic relationships between *PyDes6* (*Pythium* sp. BCC53698) and ∆^6^-desaturase genes of other organisms. Bootstrap values from 1000 replicates test are shown at individual nodes. Abbreviations of other desaturase genes in individual organisms: PciDES6, *P. citrophthora*; PsoDES6, *P.sojae*; PinDES6, *P. infestans*; PirDES6, *P. irregular*; PspDES6, *P. splendens*; AlDES6, *Albugo laibachii*; NoDES6, *N. oculata*; EsDES6, *Ectocarpus siliculosus*; TpDES6, *Thalassiosira pseudonana*; PtDES6, *P. tricornutum*; MaDES6, *M. alpina*; RsDES6, *Rhizopus stolonifer*; McDES6, *Mucor circinelloides*; MrDES6, *M. rouxii*; MpDES6, *Marchantia polymorpha*; PpDES6, *Physcomitrella patens*; BoDES6, *B. officinalis*. **Figure S3.** Growth of the recombinant yeast strains carrying the empty vector (pYES2), *PyDes6* and *MPyDes6* genes. All strains were cultivated in SD medium containing 20 g/l of raffinose for 96 h. Cell growth is represented in terms of dry cell weight (grey bar) and cell density at OD_600_ (upward diagonals bar). (DOCX 1439 kb)

## Abbreviations

ALA: α-Linolenic acid
ARA: Arachidonic acid
BCC: BIOTEC culture collection
BLAST: Basic local alignment sequencing tool
CAI: Codon adaptation index
EPA: Eicosapentaenoic acid
FAMEs: Fatty acid methyl esters
GC: Gas chromatography
GLA: γ-Linolenic acid
LA: Linoleic acid
LC-PUFA: Long-chain polyunsaturated fatty acids
RACE: Rapid amplification of cDNA ends
STA: Stearidonic acid.
