# Supplementary material for: The codon-optimized Δ6-desaturase gene of Pythium sp. as an empowering tool for engineering n3/n6 polyunsaturated fatty acid biosynthesis
Source: BMC Biotechnol. 2015 Sep 15;15:82. doi: 10.1186/s12896-015-0200-6 (PMC4570148; doi:10.1186/s12896-015-0200-6)
Supplement: Additional file 1: Figure S1. — Comparison of deduced amino acid sequences between PyDes6 of Pythium sp. BCC53698 and other ∆6-desaturases, including P. infestans (PinDES6), P. splendens (PspDES6), N. oculata (NoDES6), M. alpina (MaDES6), M. rouxii (MrDES6) and Borage officinalis (BoDES6). The conserved histidine boxes and cytochrome b 5 heme-binding motif are underlined and boxed, respectively. Figure S2. Phylogenetic relationships between PyDes6 (Pythium sp. BCC53698) and ∆6-desaturase genes of other organisms. Bootstrap values from 1000 replicates test are shown at individual nodes. Abbreviations of other desaturase genes in individual organisms: PciDES6, P. citrophthora; PsoDES6, P.sojae; PinDES6, P. infestans; PirDES6, P. irregular; PspDES6, P. splendens; AlDES6, Albugo laibachii; NoDES6, N. oculata; EsDES6, Ectocarpus siliculosus; TpDES6, Thalassiosira pseudonana; PtDES6, P. tricornutum; MaDES6, M. alpina; RsDES6, Rhizopus stolonifer; McDES6, Mucor circinelloides; MrDES6, M. rouxii; MpDES6, Marchantia polymorpha; PpDES6, Physcomitrella patens; BoDES6, B. officinalis. Figure S3. Growth of the recombinant yeast strains carrying the empty vector (pYES2), PyDes6 and MPyDes6 genes. All strains were cultivated in SD medium containing 20 g/l of raffinose for 96 h. Cell growth is represented in terms of dry cell weight (grey bar) and cell density at OD600 (upward diagonals bar). (DOCX 1439 kb) [file 12896_2015_200_MOESM1_ESM.docx]

**Additional file 1:**


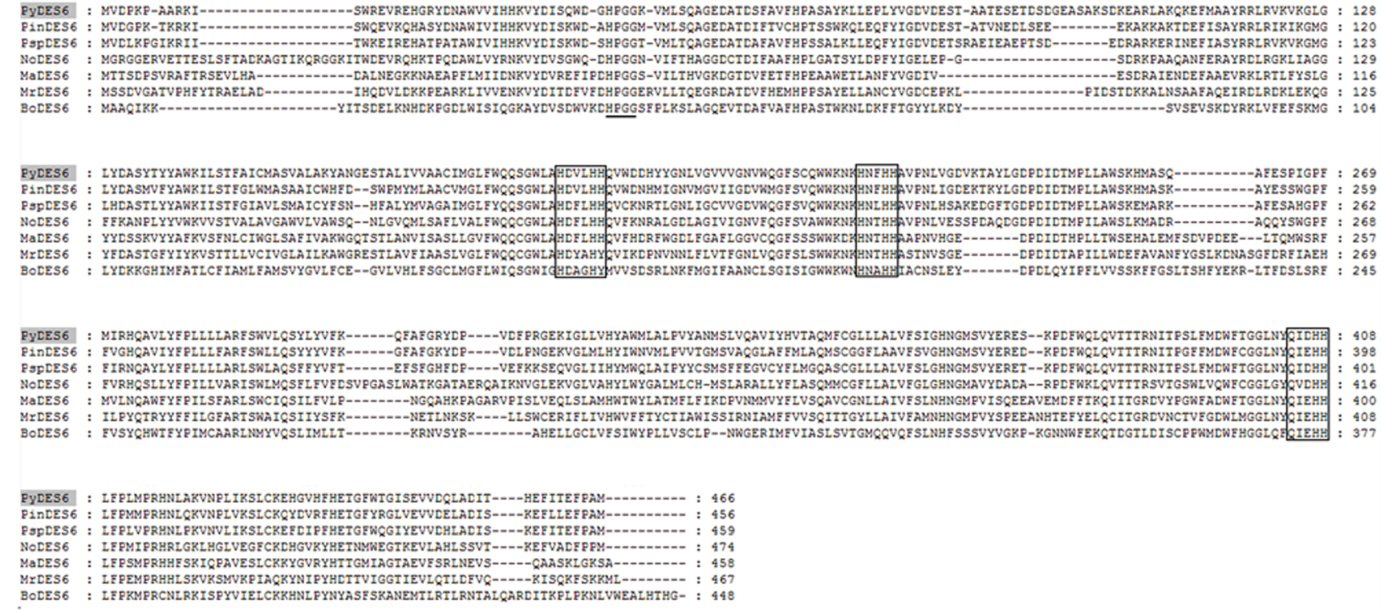


**Figure S1 Comparison of amino acid sequences between PyDES6 of *Pythium* sp. BCC53698 and other Δ^6^-desaturases**, including *P. infestans* (PinDES6), *P. splendens* (PspDES6), *N. oculata* (NoDES6), *M. alpina*(MaDES6)*, M. rouxii* (MrDES6) and *Borage officinalis* (BoDES6). The conserved histidine boxes and cytochrome *b*_5_ heme-binding motif are underlined and boxed, respectively.


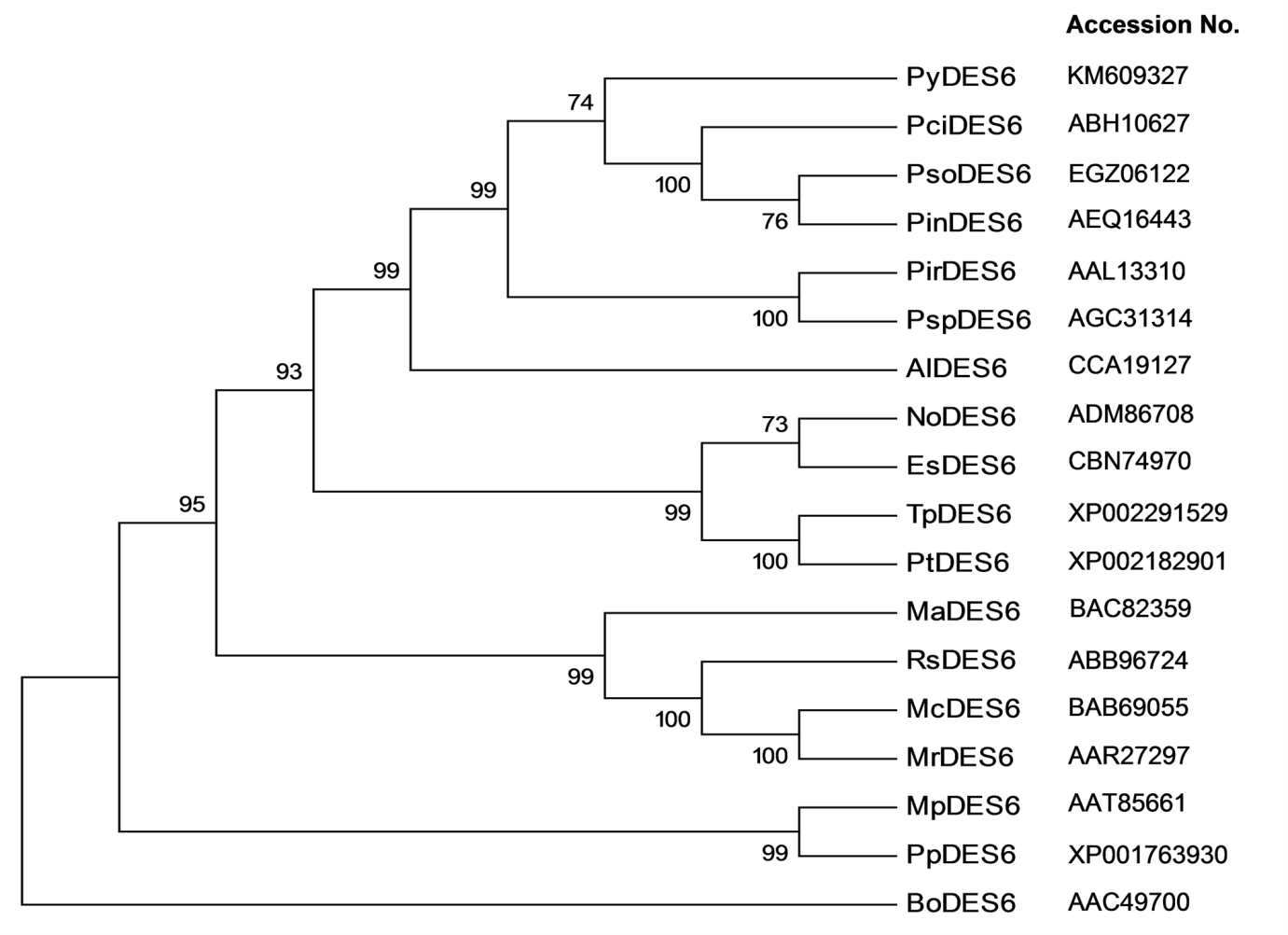


**Figure S2 Phylogenetic relationships between PyDES6 (*Pythium* sp. BCC53698) and Δ^6^-desaturase from other organisms.** Bootstrap values from 1,000 replicates test are shown at individual nodes. Abbreviations of other desaturase genes in individual organisms: PciDES6,*P. citrophthora*; PsoDES6, *P.sojae*; PinDES6, *P. infestans*; PirDES6, *P. irregular*; PspDES6, *P. splendens*; AlDES6, *Albugo laibachii*; NoDES6, *N. oculata*; EsDES6, *Ectocarpus siliculosus*; TpDES6, *Thalassiosira pseudonana*; PtDES6, *P. tricornutum*; MaDES6, *M. alpina*; RsDES6, *Rhizopus stolonifer*; McDES6, *Mucor circinelloides*; MrDES6, *M. rouxii*; MpDES6, *Marchantia polymorpha*; PpDES6, *Physcomitrella patens*; BoDES6, *B. officinalis*.


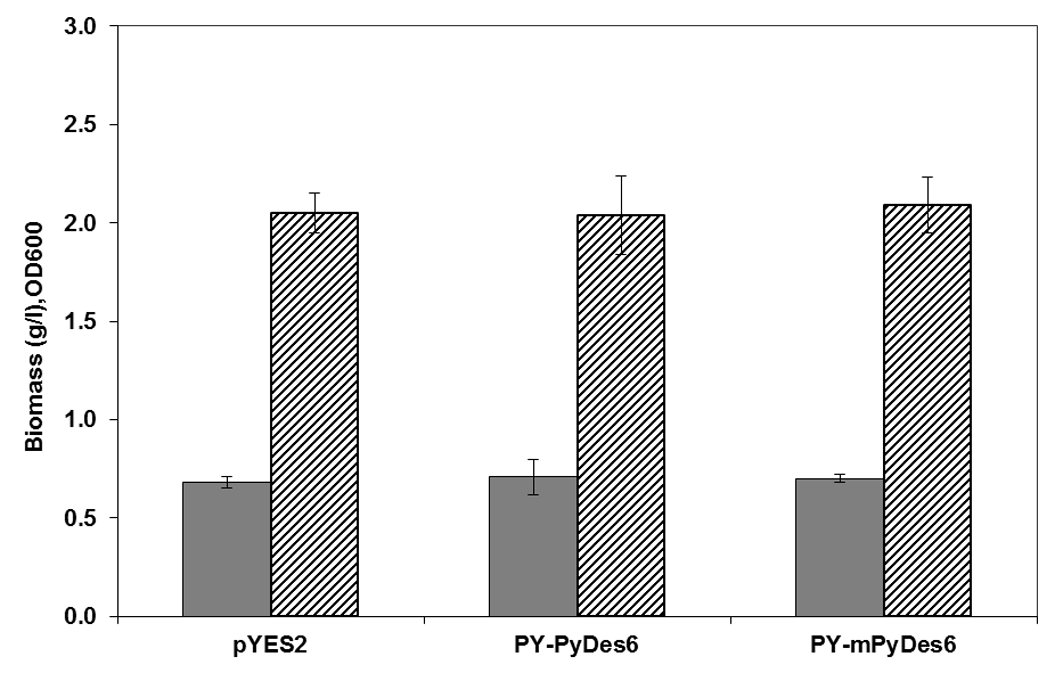


**Figure S3 Growth of the recombinant yeast strains carrying the empty vector (pYES2), *PyDes6* and *MPyDes6* genes.** All strains were cultivated in SD medium (SD) containing 20 g/l of raffinose for 96 hrs. Cell growth is represented in terms of dry cell weight (grey bar) and cell density at OD_600_ (upward diagonals bar).
